# Sex work stigma and non-disclosure to health care providers: data from a large RDS study among FSW in Brazil

**DOI:** 10.1186/s12914-019-0193-7

**Published:** 2019-03-05

**Authors:** Inês Dourado, Mark Drew Crosland Guimarães, Giseli Nogueira Damacena, Laio Magno, Paulo Roberto Borges de Souza Júnior, Celia Landmann Szwarcwald, The Brazilian FSW Group, Celia Landmann Szwarcwald, Paulo Roberto Borges de Souza Júnior, Orlando C. Ferreira, Giseli Nogueira Damacena, Neide Gravato da Silva, Rita Bacuri, Helena Brigido, Hermelinda Maia Macena, Ana Maria de Brito, Inês Dourado, Mark Drew Crosland Guimarães, Wanessa da Silva de Almeida, Alexandre Grangeiro, Carla Gianna Luppi, Karin Regina Luhm, Isete Maria Stella, Adriana Varela Espinola, Tânia Varela, Francisca Sueli da Silva

**Affiliations:** 1 Collective Health Institute, Federal University of Bahia, Av. Basílio da Gama, s/n, Campus Universitário do Canela, Salvador, Bahia 40110-040 Brazil; 2 Federal University of Minas Gerais, Belo Horizonte, Minas Gerais Brazil; 3 Institute of Scientific Communication and Information on Public Health of Oswaldo Cruz Foundation, Rio de Janeiro, Brazil; 4 Department of Life Sciences, Bahia State University, Campus 1, Salvador, Bahia Brazil

**Keywords:** Female sex worker, human rights, stigma and discrimination, health care, RDS, Brazil

## Abstract

**Background:**

Stigma in health services may be detrimental to health seeking attitudes and practices. This study investigates non-disclosure of sex work to health care providers among female sex workers (FSW) in Brazil and its association with the utilization of health care services.

**Methods:**

This study used cross-sectional respondent-driven sampling, carried out in 12 Brazilian cities to identify HIV risk behaviors among FSW. We first assessed statistical associations of sociodemographic, human right violations, health service access and utilization, and discrimination variables with non-disclosure of FSW status to health care providers as outcome. Secondly, we investigated the association of non-disclosure of FSW status with selected preventive health care outcomes: HIV testing, PAP smear exam, and post-exposure prophylaxis (PEP). Adjusted odds ratio with 95% confidence intervals were calculated by multivariable logistic regressions.

**Results:**

Among 4245 recruited FSW, a high percentage received free condoms (82%) but only 24.4% were counseled on STI. Most FSW used non-specialized public healthcare routinely (62.6%), but only 51.5% had a Pap smear exam in the last two years and less than 40% were tested for HIV in the last 12 months. Among FSW who engaged in risky behavior (49.6%), only 8.3% used PEP. Regarding human rights violations, approximately 15% were required to give part of their earnings to owners of workplace establishments, 38% started sex work under 18 years old and 6% were required to periodically present their HIV test results. 21.3% reported having faced discrimination in health services, and 24.3% always disclosed their FSW status. Multivariable logistic models indicated significant associations of non-disclosure on the four healthcare outcomes, with lower odds of using preventive health services among women who did not disclose their sex work status, even after controlling for age, educational level, NGO affiliation, and type of health care routinely used.

**Conclusions:**

Our results indicate that sex work stigmatization within health services may be one of the main barriers to STI control and HIV response among FSW. It is essential to combat stigmatization and discrimination against FSW in health services to guarantee the appropriate uptake of preventive services available in the public health system in Brazil.

## Background

Since the beginning of the AIDS epidemic, female sex workers (FSW) have been recognized as a population with high vulnerability to HIV infection stemming from individual and interpersonal factors, including biologic (e.g. co-infection with other sexually transmitted infections (STI) [1]), behavioral attitudes and practices (e.g. higher pay for riskier sex acts such as unprotected sex and drug use), and structural factors (e.g. poor socioeconomic conditions, criminalization of sex work, residential instability and violence resulting from their work) [2–6]. Furthermore, stigma and discrimination are important barriers which potentially hamper access to and use of health services [7–9], especially due to fear of public exposure and consequent negative attitudes of service providers [10].

The human rights approach proposes that “the provision of health services should be ensured to all population groups on the basis of equality and freedom from discrimination, paying particular attention to vulnerable and marginalized groups” [11], which include FSW. In this regard, governments have an obligation to protect and fulfill the human rights of their vulnerable populations by promoting equitable access to health services and adopting appropriate legislative, administrative, budgetary, judicial, promotional and other measures towards the full realization of human rights [11].

Access to the publicly funded health system in Brazil was established as a universal right of citizens and a responsibility of the State under the federal constitution. The core principles of the Brazilian National Health System or *Sistema Único de Saúde* (SUS) ─ integrality (integrated prevention, treatment and care), equity, public accountability and funding ─ resulted from a long period of advocacy for governmental responsibility for health promotion [12, 13]. Despite these principles, many FSW suffer barriers to access health services because of stigma and discrimination related to the nature of their work.

In Brazil, it is estimated that 0.8% of the female population from 15 to 49 years of age have engaged in paid sex, accounting for approximately a half million women [14]. Although prostitution in Brazil is not considered a crime under Brazilian law [15], except if minors or sexual exploitation is involved, FSW constantly experience discrimination and condemnatory, moralist and punitive attitudes [16], along with other human rights violations such as violence and harassment, usually perpetrated by partners and the police. Furthermore, situations of discrimination against women at health care settings due to social class, lack of money, race and gender have been reported in population-based Brazilian studies [17, 18], with potential adverse health outcomes [19].

Stigma and discrimination have also been pointed out as fundamental causes of population health inequalities [20] and key barriers to health access for sex workers [21–24] mainly due to fear of discrimination in health services [9, 25, 26]. In order to avoid discriminatory experiences FSW may consciously adopt a form of social invisibility by not disclosing their sex work status to health care providers, thus potentially overcoming barriers to health care [27].

To our knowledge, previous studies have not investigated associations between anticipated stigma and access and uptake of health services in Brazil. The objective of this paper was to investigate non-disclosure of FSW status to health care providers and its association with access and uptake of preventive health care services (Pap smear, HIV testing, awareness and use of PEP).

## Methods

### Study design

This study reports results of a cross-sectional Biological and Behavioral Surveillance Survey (BBSS) among FSW conducted in 12 Brazilian cities, in 2016. Cities were defined, a priori*,* by the Department of STI/HIV/AIDS and Viral Hepatitis, Ministry of Health (DIAHV/MoH) representing the five regions of Brazil (São Paulo, Belo Horizonte and Rio de Janeiro – Southeast Region; Curitiba and Porto Alegre – South Region; Brasília and Campo Grande – Central-West Region; Fortaleza, Recife and Salvador – Northeast Region; and, Belém and Manaus – North Region). Co-Investigators were responsible for conducting the study in each of the 12 cities and their names are listed under the Brazilian FSW Group.

Participants were recruited using respondent-driven sampling (RDS), and data was collected on HIV risk behavior practices, access to health services and situations of stigma and discrimination, among others. The research project was approved by the Oswaldo Cruz Foundation Ethics Committee (Protocol 1.338.989).

The sample size was set at 350 FSW in each city. Women were eligible to participate in the study if they met the following inclusion criteria: age 18 years old or over; report working as a sex worker in one of the cities of the study; have had at least one sexual transaction in exchange for money in the past four months; present a valid RDS coupon to participate; and, signed written informed consent. RDS was chosen as the most appropriate method among available alternatives for reasons that included the hidden nature of FSW social networks [28–30].

Fieldwork was conducted in health services located in the 12 cities. As required by the RDS method, six to eight initial participants in each city - called seeds - were chosen purposively, following formative qualitative research with focus groups with local FSW leaders, non-governmental organizations (NGO), potential participants, and researchers. Each seed received three coupons to distribute to other sex workers from her social network. The recruits of the seeds in the survey were considered the first wave of the study. After participating in the interview, each participant received three additional coupons to distribute to their peers and this process was repeated until the sample size was achieved in each site.

RDS requires a system of primary and secondary incentives. The primary incentive in this study was a gift (makeup products), payment for lunch and transportation in addition to a reimbursement for their time lost from work (approximately US$15.00). The secondary incentive was a payment of US$10.00 for each recruited person who participated in the study.

### Data Collection

The questionnaire included modules on: sociodemographic characteristics and information related to professional activity, knowledge about HIV transmission, sexual behavior, previous HIV test - lifetime and in the last year, STI history, use of alcohol and illicit drugs, access to prevention activities and health services, discrimination and violence. The questionnaire was designed for tablet computers and could be self-administered according to the participant’s desire and readiness. Tests for HIV, syphilis, and hepatitis B and C were conducted by standard rapid tests using peripheral venous blood collection, according to protocols recommended by the Brazilian Ministry of Health [31]. All tests occurred before the interview and all participants received pre- and post-test counseling. Participants who tested positive in any one of the rapid tests received additional post-test counseling, both to address the psychological impact and to encourage partner notification, and they were also referred to public health services for follow-up.

### Study Variables

For this analysis, we defined four main groups of selected variables: socio-demographics, human rights violation indicators, access and uptake of health services indicators, disclosure of FSW status to health care providers and discrimination. Educational level was based on current Brazilian classification; race/skin color was categorized as white, black, brown or other (yellow, indigenous). FSW belonging to non-governmental organizations (NGO) was assessed as self-reported. Although prostitution is legal in Brazil for women aged 18 or over, the exploitation of sex workers is not. Thus, the following factors were taken as indicators of human rights violations against FSW as these are prohibited by labor laws in Brazil: a) requirement to give any percentage of their earnings to the owner of the establishment; b) requirement to give any percentage of their earnings to a pimp; c) sex work debut under 18 years old; d) compulsory submission of HIV test results to a supervisor in the workplace. Regarding access to uptake of health services, we considered the following indicators: a) use of a regular healthcare service (none, primary health care or specialized public health care services, and private services); b) had Pap smear exam in the last year, one to less than two years ago, three or more years ago, or never; c) HIV testing in the last year, one or more years ago, or never; d) awareness of post-exposure prophylaxis (PEP); e) PEP use if exposed to HIV risk over the past six months with PEP indication, i.e., condom breaking, bursting or sliding at least once; having been forced to have sex without a condom; and, client removed condom during sex without the FSW permission. Finally, as indicator of sex work disclosure and discrimination, we included: a) disclosure of FSW status to health care providers based on the following question: “When you go to a health service, do you disclose your sex work status to health care providers?” and it was categorized as always, sometimes, or never; and, b) perception of discrimination in the health services based on the question: “Have you ever felt discriminated against or treated worse than other people in the health services for being a FSW?” which was categorized as yes or no. Additionally, for those who answered never having had a Pap smear or HIV testing, we inquired whether this was due to shame of disclosing their FSW status, as proposed by UNAIDS [32].

### Data analysis

Statistical methods appropriate for RDS design were used for data analysis, taking into account the dependence among observations resulting from the recruitment chains, and the unequal probabilities of selection resulting from the different sizes of networks of each participant. Seeds were excluded from this analysis considering that they were not recruited by their peers and did not contribute to the social network size used for weighting [33]. Each city composed a stratum, and each group of women recruited by the same FSW composed a cluster. The weighting was based on the inverse probability of selection proportional to the size of the network of each participant [33]. In this study, the question used to measure the network size of each participant and the resulting weighting was: “How many sex workers who work here in this city do you know personally?”. Network sizes were limited to the range 3–150, i.e., network sizes of one and two were recoded to three and higher values to 150.

Overall descriptive analysis was conducted, and analyses of associations were divided into two parts. We initially assessed potential statistical associations of sociodemographics, human rights violations, access and uptake of health services with non-disclosure of FSW status to health care providers. Those who answered “never disclosing” were compared to those who always or sometimes disclosed their FSW status. Adjusted odds ratios (AOR) with 95% confidence intervals were then estimated using multivariable logistic regression modeling of those variables statistically significant (*p* < 0.05) in the univariable assessment.

In the second part, we assessed the association of non-disclosure, now as potential predictor with two levels (“never disclosing” and always/sometimes disclosed their FSW status), on four indicators of preventive health care (1. Pap smear exam in the last two years, 2. HIV testing in the last year, 3. awareness of PEP, and 4. PEP use after exposure to risk). We chose these indicators as they measure access to important health care to prevent HIV and cervical cancer mortality among women. For each one of these four indicators, we calculated the AOR of non-disclosure of FSW status to health care providers after controlling for age, educational level, belonging to a FSW NGO, type of health service routinely used, and perception of discrimination.

## Results

Among 4328 FSW recruited into the study, 4245 women 18 years and over were included in this analysis, after excluding the seeds. The average number of FSW recruited by each participant was 2.6. Approximately 50.0% were younger than 30 years old, 48.0% had not completed high school, 52.3% self-reported as brown (someone from a mixed race), and only 8.0% belonged to an NGO (Table 1). As to human rights violations, approximately 15.0% had to give part of their earnings to the establishment owner or to the pimp, and 38.0% started sex work under 18 years old. Compulsory submission of an HIV test results to a supervisor in the workplace was reported by 6.3%.

**Table 1:** Socio-demographics, social rights, human rights violations, and health care indicators among FSW. Brazil, 2016

Variables	n	Percentage (%)	95% CI
Socio-demographics			
Age group			
18–29	2110	49.7	47.6–51.8
30–39	1118	26.3	24.6–28.1
40–49	650	15.3	13.9–16.8
50+	367	8.6	7.6–9.8
Educational level			
Incomplete elementary school	626	14.9	13.5–16.4
Incomplete high school	1386	32.9	31.1–34.8
Complete high school	1096	26.0	24.3–27.8
Incomplete college or more	1104	26.2	24.5–28.0
Belonging to FSW NGO			
Yes	326	7.8	6.8–8.9
No	3859	92.2	91.1–93.2
Human Rights violations			
Required to give a percentage of their earnings to owner of the establishment			
Yes	524	12.5	11.1–13.9
No	3681	87.5	86.1–88.9
Required to give a percentage of their earnings to a pimp			
Yes	175	4.2	3.4–5.0
No	4026	95.8	95.0–96.6
Started sex work under 18 years old			
Yes	1628	38.7	36.7–40.7
No	2580	61.3	59.3–63.3
The workplace required periodic HIV testing			
Yes	265	6.3	5.4–7.4
No	3934	93.7	92.6–94.6
Health service utilization			
Regular source of care			
Does not have one	1193	29.0	27.2–30.9
Primary health care	2588	62.9	60.9–64.9
Public specialized health care	101	2.5	1.9–3.2
Private health care	230	5.6	4.8–6.5
Had Pap smear exam in			
Less than 1 year ago	1315	31.5	29.7–33.3
1 to less 2 years ago	837	20.0	18.5–21.6
2 to less than 3 years ago	454	10.9	9.6–12.3
3 years or more ago	772	18.5	17.0–20.1
Never	802	19.2	17.7–20.8
Had HIV testing in			
Less than 1 year ago	1642	39.3	37.4–41.2
1 year or more ago	1592	38.1	36.2–40.0
Never	946	22.6	21.0–24.3
Awareness of PEP^a^			
Yes	1299	31.1	29.3–33.3
No	2880	68.9	67.0–70.7
Risk exposure in the past 6 months with PEP indication^b^			
Yes	2079	49.6	47.6–51.5
No	2116	50.4	48.5–52.4
PEP use among FSW exposed to risk			
Yes	172	8.3	6.8–10.0
No	1907	91.7	90.0–93.2

^a^PEP: post exposure prophylaxis

^b^ Condom broke, burst, slid at least once or was forced to have sex without a condom or the condom was removed during sex without permission at least once over the past 6 months

As access to and uptake of health services, most FSW used primary health care routinely (62.6%), 5.6% used private health care, and only 2.5% used publicly funded specialized health care. A large proportion (29.0%) did not have a regular source of health services. The proportion of FSW who had a Pap smear exam in the past two years was close to 50%. HIV testing at least once in their lifetime was high (77.4%), but less than 40% had been tested over the last 12 months. Awareness of PEP was reported by 31% and, among FSW who reported engaging in risky behavior over the past six months with PEP indication (49.6%), only 8.3% used PEP (Table 1).

More than half of the women never disclosed their FSW status (51.5%) to health care providers and 21% felt discriminated against or were treated worse than other people for being FSW. Furthermore, 14.7% and 12.1% reported shame of revealing their FSW status as the reason for never having had a Pap smear exam or HIV testing, respectively (Table 2).

**Table 2:** Disclosure of FSW status and perception of discrimination in health services. Brazil, 2016

Indicators		n	Percentage (%)	95% CI
Disclosure of FSW status to a health care staff	Always	1014	24.3	22.7–25.9
	Not always	1012	24.2	22.6–25.9
	Never	2153	51.5	49.6–53.5
Perception of discrimination or were treated worse in health services for being a FSW	Yes	893	21.3	19.8–23.0
	No	3294	78.7	77.0–80.2
Had never had a Pap smear because of shame of disclosing FSW status	Yes	100	14.7	11.4–18.7
	No	584	85.3	81.3–88.6
Had never tested for HIV because of shame of disclosing FSW status	Yes	112	12.1	9.4–15.4
	No	816	87.9	84.6–90.6

Results of the initial univariable and multivariable analyses of non-disclosure of FSW status to health care providers are seen in Tables 3 and 4 respectively. The multivariable analysis shows statistically significant associations of age (younger women) with non-disclosure of FSW status to health care providers. Educational level (lower education), and belonging to FSW NGO were associated with disclosure of FSW status to health care providers. In addition, the odds of non-disclosure were higher among FSW who did not have a regular source of health care (OR = 2.36) or among those who used private health care (OR = 1.99), as compared to public specialized health care (Table 3).

**Table 3:** Factors associated to non-disclosure of FSW status to health care staff

Variables		Non-disclosure of FSW status to health care staff					
		OR	95% CI	*p*-value	Adjusted OR	95% CI	*p*-value
Age	18–29	1.56	1.22–2.00	< 0.001	1.32	1.02–1.72	0.034
	30–39	1.42	1.06–1.90	0.018	1.23	0.91–1.66	0.180
	40–49	1.17	0.91–1.50	0.219	1.04	0.80–1.35	0.749
	50+	1.00	–	–	1.00	–	–
Educational level	Incomplete elementary school	0.57	0.43–0.76	< 0.001	0.65	0.48–0.88	0.005
	Incomplete high school	0.69	0.54–0.87	0.002	0.70	0.55–0.90	0.006
	Complete high school	0.99	0.77–1.28	0.964	0.99	0.76–1.27	0.912
	Incomplete college or more	1.00	–	–	1.00	–	–
Belonging to FSW-NGO	Yes	0.66	0.50–0.88	0.005	0.72	0.53–0.98	0.036
	No	1.00	–	–	1.00	–	–
Regular source of care	Does not have one	2.82	1.62–4.89	< 0.001	2.36	1.31–4.24	0.004
	Private care	2.51	1.32–4.76	0.005	1.99	1.02–3.91	0.045
	Public specialized care	1.00	–	–	1.00	–	–
	Public not specialized care	1.47	0.87–2.50	0.151	1.26	0.72–2.22	0.414

**Table 4:** Association of non-disclosure of FSW status to health care staff with preventive health care, PEP awareness and use after controlling for selected variables. Brazil, 2016

Outcomes	Non-disclosure of FSW status to health care staff		
	Adjusted OR^a^	95% CI	p-value
Pap smear exam <2y ago	0.82	0.67–0.99	0.040
HIV testing < 1 y ago	0.80	0.67–0.97	0.020
Awareness of PEP	0.72	0.59–0.88	0.001
PEP use among FSW exposed to risk	0.39	0.26–0.60	< 0.001

^a^Adjusted for age, educational level, belonging to NGO, and regular source of care

Finally, the association of non-disclosure of FSW status to health care providers with preventive care indicators is presented in Table 4, after adjusting for age, educational level, belonging to FSW NGO, and regular source of health care. Non-disclosure of FSW status to health care providers was statistically associated (*p* < 0.05) with all four indicators with lower odds of using preventive health care among women who did not disclose the FSW status to health care providers compared to FSW who always revealed their status. Similar results were found for awareness of PEP and PEP use when exposed to HIV risk.

## Discussion

The Brazilian state is signatory to all international agreements that directly or indirectly guarantee women’s human rights, as well as the elimination of all forms of discrimination and violence based on gender. Although prostitution is a non-criminalized occupation in Brazil, there are many violations of female sex workers’ human rights. Currently, the requirement to pay back part of their earnings to third parties, as our results indicate, is considered exploitation of sex work and this is not legally permitted.

The comparison of our current analysis with those found in a previous study carried out by us in 2009 with a similar methodology [34] shows that engaging in paid sex is starting earlier. While in 2009 the proportion of girls who engaged in paid sex under the age of 18 years old (a sexual exploitation of minors) was 28%, in 2016 this percentage rose to 39%, with a worrisome 13% starting at age 14 years old or less.

Compulsory submission of test results to a supervisor in the workplace or pimps is an additional violation of FSW human rights, as compulsory assessment as well as dismissal from any employment due to HIV infection are forbidden by law [6]. Since there is no official job contract, in general FSW are merely removed from the workplace, without any social guarantee, such as unemployment insurance. It should be noted that 70% of the FSW interviewed in the present study favored sex work regulations and a possible formal contract providing legal job guarantees. But, only 2% reported having such a work contract.

The implementation of the Brazilian Health System represented an important change in the organization of health services in the country, especially with the strengthening of primary health care (PHC). The Family Health Program is the main approach to provide primary care services within Brazil’s national health system in an effort to also reach underserved communities [35]. The results of this study confirm the use of PHC units as the main source of care among FSW, with only a small portion seeking specialized care services. Our results indicate that non-disclosure of FSW status to health care providers was associated with poor uptake of preventive exams, such as Pap smear exam in the last 2 years and HIV testing in the last 12 months, despite availability of testing and screening services free of charge in PCH units.

We speculate that more preventive health services are offered to women who are considered “at higher risk for IST” such as FSW [36]. Because of the nature of the study design we cannot be certain of the direction of this association, the interpretation could also be that once FSW reveal their status, perception of discrimination occurs. This result should be further explored in other studies. Nevertheless, health care guidelines should recommend that health authorities should ensure that health providers be trained to deal with the stigma and discrimination associated with sex work.

As previously pointed out, stigma within health services may be detrimental to health seeking behaviors and constitute one of the main barriers to the HIV response [25, 26, 37, 38]. Furthermore, in the national HIV/AIDS surveillance system, the occupation of each reported HIV case is classified according to the Brazilian Occupation Classification. However, in spite of sex work being part of the list of occupations, it is seldom stated as the occupation in the surveillance system, greatly restricting analysis of AIDS incidence and HIV prevalence in this population group and limiting monitoring of interventions focused on FSW.

Anticipated stigma hampers access to health care, but this phenomenon is a structural problem in Brazilian society, which does not recognize the rights of FSW. The government bodies must adopt appropriate legislative, administrative, judicial, promotional and other measures to protect sex workers’ human rights. Their ability to organize is a strategy against HIV/AIDS based on organization of sex workers to conduct programs to achieve most effective HIV prevention outcomes and human rights. It is known to be effective for reductions in HIV and STI outcomes and increases in consistent condom use with clients [39–42]. However, there are structural barriers to implement this strategy because of stigma, discrimination and violence, and especially because of less and less financial resources from government and donors [43].

Regarding the use of PEP, the proportion of FSW who used PEP was rather small, less than 10%, although half had been exposed to an HIV risk that should have triggered the use of PEP. In view of our current results showing that both awareness of PEP as well as its use are associated with non-disclosure of FSW status to health care providers, and that pre-exposure prophylaxis (PrEP) has recently been introduced in Brazil, it is urgent to develop public health policies which enable disclosure of sex work status to health care providers. Improvements have been achieved in the distribution of free condoms, regardless of the amount requested or reasons for large quantities with no-questions-asked policy (70% received free condoms in satisfactory amounts). At the same time, it is essential to combat stigmatization and discrimination against FSW from health care providers in order to guarantee the appropriate uptake of preventive methods and treatment already available in the Brazilian public health system. Programs that foster inclusion of marginalized groups such as adolescent girls who engage in paid sex are also urgently needed.

## Limitations

This was a cross-sectional RDS study. Although our analysis took into consideration the complex design of the study, potential homophily, size and duration of the recruitment chains may still hamper representativeness of the target population of interest. Potential correlations among some of our variables, e.g. perception of discrimination and disclosure, are also of concern for our multivariable modeling, with unclear direction of the association. In addition, since we considered only a limited number of potential predictors, unknown confounding can still be present.

## Conclusions

Despite universal access to health services in the Brazilian public health system, the results of this study show that sex work stigmatization and discrimination in health services may be major barriers to the maximal effectiveness of prevention and care actions for FSW in Brazil. Interventions targeting stigma and discrimination against FSW in health care services are urgently needed to increase access to health services.

For a sustained HIV response, efforts must focus on building awareness of FSW health care rights and on addressing barriers to health care access and utilization, such as reducing sex work-related stigma, aiming at transforming health services into safe environments where FSW can disclose their status without fear of facing discrimination. Moreover, community empowerment-based HIV responses must enable sex workers to develop and implement public health actions themselves, and to encourage recognition of sex work as legitimate work in society. Among adolescent girls less than 18 years’ old who engage in paid sex, clear strategies are needed to reach this priority group despite the illicitness and social invisibility of adolescent girls.

## Abbreviations

AIDS: Acquired Immune Deficiency Syndrome
DIAHV/MoH: Department of STI/AIDS and Viral Hepatitis, Ministry of Health
FSW: Female Sex Workers
HIV: Human Immunodeficiency Virus
NGO: Non-Governmental Organization
OR: Odds ratios
PEP: Post-exposure prophylaxis
PHC: primary health care
PrEP: Pre-exposure prophylaxis
RDS: Respondent Driven Sampling
STI: Sexually transmitted infections
SUS: Brazilian National Health System
UNAIDS: Joint United Nations Programme on HIV/AIDS
